# Wide belt sowing improves the grain yield of bread wheat by maintaining grain weight at the backdrop of increases in spike number

**DOI:** 10.3389/fpls.2022.992772

**Published:** 2022-08-18

**Authors:** Xiu Zhang, Yifan Hua, Yunjing Liu, Mingrong He, Zhengchun Ju, Xinglong Dai

**Affiliations:** ^1^State Key Laboratory of Crop Biology, Ministry of Science and Technology, Key Laboratory of Crop Ecophysiology and Farming System, Ministry of Agriculture and Rural of Affairs, Agronomy College of Shandong Agricultural University, Tai’an, China; ^2^Shandong Provincial Department of Agriculture and Rural Affairs, Shandong Agricultural Technology Extension Center, Jinan, China

**Keywords:** bread wheat, wide belt sowing, antioxidant enzyme activity, canopy apparent photosynthesis, dry matter production, grain yield

## Abstract

Increasing the seeding belt width from 2 to 3 cm (conventional drilling sowing, CD) to 8–10 cm (wide belt sowing, WB) can markedly improve the grain yield of bread wheat. However, there are insufficient data to explain how WB affects dry matter (DM) remobilization, pre- and post-anthesis production, and ultimately grain weight and grain yield. In the present study, four bread wheat cultivars (Jimai44, Taishan27, Gaoyou5766, and Zhouyuan9369) with similar phenology characteristic were selected as experimental materials and two sowing patterns (CD and WB) were applied during the 2018–2019 and 2019–2020 growing seasons, to investigate the effects of sowing pattern on grain yield and its components of bread wheat. The results showed that WB increased the post-anthesis rate of canopy apparent photosynthesis (CAP) in comparison with CD, by 19.73–133.68%, across the two seasons and four bread wheat cultivars. Furthermore, WB significantly increased the activities of superoxide dismutase, peroxidase, and catalase, and decreased the malondialdehyde content of the flag and penultimate leaf, thereby extending the duration of the high-value CAP period by 1.95–2.51 days. The improved rate and duration of CAP in WB led to an increase in post-anthesis DM production of 13.33–23.58%, thus ensuring DM distribution to the grain of each bread wheat cultivar. Consequently, in WB, the grain weight was maintained, the grain yield was increased markedly by 9.65–15.80%, at the backdrop of increases in spike number and in turn grain number per unit area. In summary, WB could be applied widely to obtain a high yield of bread wheat.

## Introduction

High-quality bread wheat is consumed every day all over the world and utilized to make flour for bread, noodles, and other foods (Rossmann et al., 2020; Guo et al., 2021; Liu et al., 2021). Because of its advantages in terms of product quality, taste, and nutrition, there is an increasing demand for bread wheat. More than 9 billion kilograms of bread products are produced every year, and global bread consumption is approximately 70 kg person^–1^ year^–1^ (Wang et al., 2021), consequently, there is a great need to improve bread wheat production. Due to the increasing population and decreasing planting area (Food and Agricultural Organization of the United Nations [FAO], 2020), improving total grain yield by increasing the grain yield of wheat per unit area is key for ensuring an adequate food supply and reducing hunger (Zheng et al., 2011; Sun et al., 2014).

Studies in the United Kingdom (Shearman et al., 2005), Australia (Sadras and Lawson, 2011), China (Xiao et al., 2012), Brazil (Beche et al., 2014), and northwest Mexico (Aisawi et al., 2015) have shown that grain yield is closely associated with dry matter (DM). In detail, during grain filling, grain accumulates DM from two sources: current assimilates transferred post-anthesis directly to the grain, and pre-anthesis assimilates redistributed from reserve pools stored in vegetative plant parts (Santiveri et al., 2004; Moradi et al., 2022).

The importance of each source of grain filling, and the contribution to grain yield, vary among genotypes, depend on the environmental conditions during the grain-filling period, and are related to agronomic measurements. In detail, assimilates remobilized pre-anthesis was driven by DM at anthesis (Fan et al., 2022), and are influenced by the transport ability of DM from vegetative organs to grain in different genotypes (Arduini et al., 2006) and under different growing conditions (Giri et al., 2019). The contributions of assimilates remobilized pre-anthesis account for 20–40% (Yan et al., 2022; Zhang et al., 2022), and the proportion can approach 60% under heat stress (Plaut et al., 2004). The contribution of post-anthesis DM to grain yield has been reported to range from 60 to 80% in wheat. In previous investigations regarding the optimization of genotypes (Zhang et al., 2016), tillage practices (Wang et al., 2020), irrigation (Li et al., 2021), nitrogen (N) fertilizer input (Ferrise et al., 2010), and seeding rate (Arduini et al., 2006), wheat yield was mostly increased by increasing post-anthesis DM production. Furthermore, post-anthesis DM production is closely related to the flag leaf and penultimate leaf owing to their important contribution to the canopy photosynthesis during grain filling (Konstanze et al., 2005; Townsend et al., 2017).

However, plant distribution in the field and plant spacing (in rows) may influence crop development and growth, and ultimately grain yield (Liu X. et al., 2020). Wide belt sowing (WB) increases the seedling belt to 8–10 cm, compared with the 2–3 cm distance used in conventional drilling sowing (CD). Expansion of the seedling belt increases the distance between neighboring plants, which reduces intraspecific competition within belts and enhances grain yield (Li et al., 2015; Fan et al., 2019; Liu X. et al., 2020; Ma and Li, 2020). Moreover, in comparison with CD, WB only requires an increase in the width of the moldboard of the furrow opener and does not increase other costs.

WB can maintain good grain quality, and higher grain yield and N use efficiency, by optimizing the coupling of the grain yield formation process with the processes of N uptake and translocation (Liu et al., 2022), this suggests that WB could be used widely for the production of bread wheat. However, there are insufficient data regarding how WB affects pre- and post-anthesis DM remobilization, production, and ultimately grain weight and grain yield.

Therefore, an experiment with four bread wheat cultivars (Gaoyou5766, Jimai44, Taishan27, and Zhouyuan9369) as experimental materials and two sowing patterns (WB and CD) was performed during the 2018–2019 and 2019–2020 growing seasons. We studied the effects of sowing pattern on flag and penultimate leaf senescence, the rate and duration of canopy apparent photosynthesis (CAP), pre- and post-anthesis DM remobilization and production, yield components, and the grain yield of bread wheat. The goal was to clarify the physiological basis of the effect of WB on the grain yield and to provide a theoretical basis for high-yield cultivation of bread wheat.

## Materials and methods

### Plant materials and growing conditions

**FIGURE 1:**
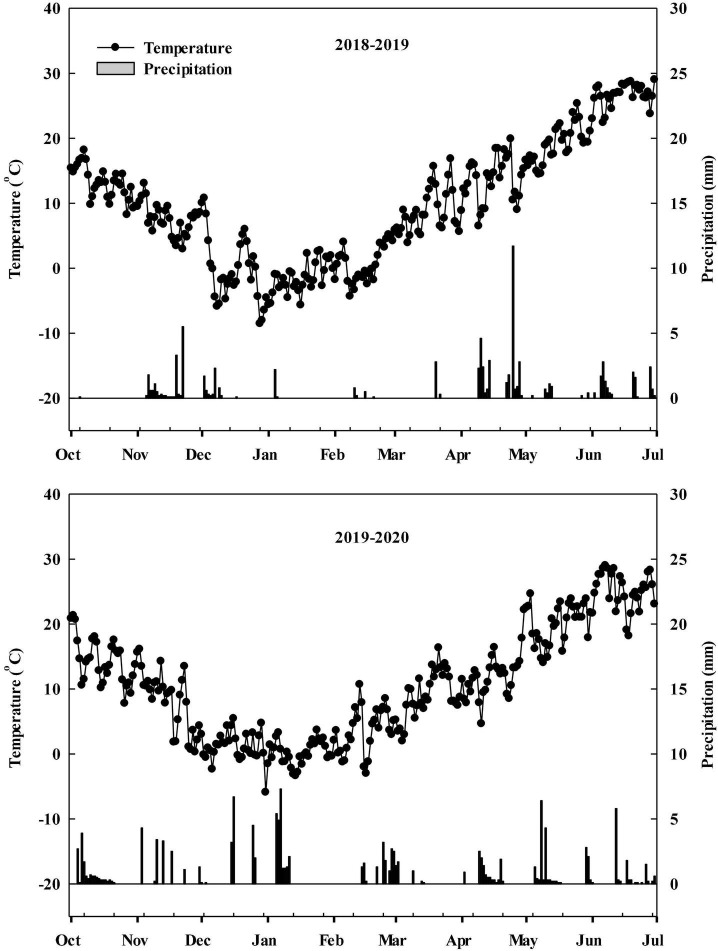
Precipitation and mean temperature during the winter wheat growth period (October to the following June) in the 2018–2019 and 2019–2020 growing seasons.

During the 2018–2019 and 2019–2020 winter wheat growing seasons, field experiments were carried out at the experimental station of the village of Dongwu (35°57′ N, 117°03′ E), Dawenkou Town, Daiyue District, Tai’an City, Shandong Province, China. Precipitation and temperature data were obtained from a meteorological station located less than 500 m from the experimental field (Figure 1). The previous crop grown in the field was summer maize, and all straw remaining on the plots had been plowed into the field for many years. The soil was characterized as sandy loam (typic Cambisol). Nutrient status of top 0–20 cm soil before seeding in 2018–2019 and 2019–2020 were shown in Supplementary Table 1.

**FIGURE 2:**
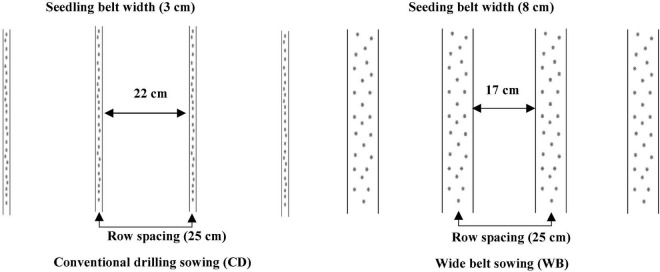
Schematic diagram of conventional drilling sowing and wide belt sowing.

Seeds of bread wheat cultivars Jimai44 (J44), Taishan27 (T27), Gaoyou5766 (G5766), and Zhouyuan9369 (Z9369) with good breading quality were sown in two sowing patterns (Figure 2): CD (seedling belt width of 2–3 cm) and WB (seedling belt width of 8–10 cm). Bread wheat J44 and G5766 were cultivars with small ears and high tillering capacity, and were sown at densities of 240 plants m^–2^, while T27 and Z9369 were cultivars with large ears and low tillering capacity, and were sown at densities of 375 plants m^–2^. The four cultivars had similar phenology characteristic and the corresponding phenology information in the two growing seasons was shown in Table 1. The experiment used a split-plot design, with the cultivar as the main plot and each sowing pattern as a sub-plot, with three replicates. The area of each plot was 36 m^2^ (24 m × 1.5 m). Before tillage, 96 kg ha^–1^ of N, 120 kg ha^–1^ of P_2_O_5_, and 120 kg ha^–1^ of K_2_O were applied; another 144 kg ha^–1^ of N was applied at the jointing stage. Irrigation (approximately 60 mm each time) was performed before wintering, at the jointing stage, and at the anthesis stage.

**TABLE 1:** The date at which bread wheat reached sowing, wintering, jointing, anthesis, and maturity stages for cultivars.

Growing season	Cultivar	Growing stage (mm/dd/yy)					Days of growing season (d)
		Sowing	Wintering	Jointing	Anthesis	Maturity	
2018–2019	J44	10/13/2018	12/14/2018	3/31/2019	5/1/2019	6/7/2019	237
	T27	10/13/2018	12/14/2018	3/31/2019	5/2/2019	6/9/2019	239
	G5766	10/13/2018	12/14/2018	3/31/2019	5/1/2019	6/7/2019	237
	Z9369	10/13/2018	12/14/2018	3/31/2019	5/2/2019	6/9/2019	239
2019–2020	J44	10/14/2019	12/13/2019	3/29/2020	4/30/2020	6/8/2020	238
	T27	10/14/2019	12/13/2019	3/29/2020	5/1/2020	6/9/2020	239
	G5766	10/14/2019	12/13/2019	3/29/2020	4/30/2020	6/8/2020	238
	Z9369	10/14/2019	12/13/2019	3/29/2020	5/1/2020	6/9/2020	239

### Grain yield and dry matter at anthesis and maturity

At maturity, grain yield was measured by harvesting all plants in a 2.0 m × 1.5 m area in each plot and adjusted to a standard moisture content of 13% (87% DM) (Dai et al., 2013). The spike number per unit area was obtained at the maturity stage. In each plot, the number of spikes from 1.5-m^2^ random sample points was counted non-destructively. The number of spikes per hectare was then calculated according to the area occupied by the sample points. The grain number per spike was determined by the average number of grains of 30 spikes. The 1,000-grain weight was recorded from the air-dried grain measured to determine yield in the plot.

Thirty single stems were sampled randomly at anthesis and maturity. The sampled plants were separated into stems, leaves, and ears at anthesis, and stems, leaves, grains, and glumes + ear rachis at maturity. All separated samples were oven dried at 70°C to constant mass, to estimate plant biomass. According to Zhang et al. (2022), the dry matter remobilization amount in vegetative organs (DMRA) was defined as the difference between the DM amount accumulated during the anthesis period (DMAA) and the DM amount accumulated in vegetative organs during the mature period (DMAM). The dry matter remobilization efficiency in vegetative organs (DMRE) was defined as the ratio of DMRA to DMAA. The total contribution rate of dry matter remobilization pre-anthesis to yield (DMRCR) was taken as the ratio of DMRA to grain dry weight. The amount of dry matter production post-anthesis (DMPA) was defined as the difference between the grain dry weight and DMRA, and the total contribution ratio of the post-anthesis DM accumulation to grain (DMACR) was 1—DMRCR.

### Canopy apparent photosynthesis

The CAP values at 5 days post-anthesis (DPA), 10 DPA, 15 DPA, 20 DPA, and 25 DPA were measured using a CO_2_ analysis system (GXH-3501; Junfang Institute of Physics and Chemistry, Beijing, China) according to the method described in Garrity et al. (1984) and Reddy et al. (1995) with some modification. The chamber was constructed from polyester film (95% light transmittance). Its dimensions of 0.70 m (length) × 0.60 m (width) × 1.20 m (height) could enclose all wheat plants within a 0.50-m row. Air was mixed in the chamber by a small built-in fan with a diameter of 30 cm.

Measurements of photosynthesis were made in the morning between 9:00 a.m. and 11:00 a.m. on clear days. The chamber was opened prior to measurements to maintain the CO_2_ concentration in the chamber at 400 ± 15 μmol mol^–1^, equal to that in the atmosphere. The wheat system was then enclosed in the chamber and exposed to full sunlight (1,250 ± 50 μmol m^–2^ s^–1^). The rapid decline in CO_2_ concentration in the chamber was recorded until there was a steady decrease between 380 and 280 μmol mol^–1^ for a duration of at least 60 s. CAP was calculated according to Chen et al. (2021) as follows:


(1) CAP=((dc/dt)×V×D)/S×10∧,6

(2) D=(44,000/ 0.082)/T,

where *d*c is the change in CO_2_ (μmol mol^–1^), *d*t is the time taken for the change in CO_2_(s), V is the volume of the leaf chamber (m^3^), S is the wheat area in the chamber (m^2^), D is the CO_2_ gas density, and T is the environmental temperature (K).

### Modeling canopy apparent photosynthesis post-anthesis

The CAP data were fitted to a three-parameter logistic regression analysis (Eqs. 3–5) according to Kaewtapee et al. (2011) as follows:


(3) Y=K/[1+ae∧](-bt),

(4) Y=′dY/dt=Kabe∧/(-bt)[1+ae∧](-bt)2

(5) Y=′′d2Y/dt2=Kab3e(-bt)∧[1-4abe(-bt)∧+a2e(-2bt)∧]/[1+ae(-bt)∧]4,

where *t* represents the number of DPA, Y represents the CAP, and K, a, and b are the three constants of the logistic model. The logistic model yielded an inverted “S” shaped curve.

Calculation of the second derivative (Y′′) provides the DPA at which the maximum CAP was attained. This maximum corresponds to the theoretical value when Y′′ = 0, that is, when


1-4abe(-bt)∧+a2e(-2bt)∧=0.

Then, we get the following:


$${\text{t}}_{1}=\left(\mathrm{lna}+1.317\right)/b,{\text{t}}_{2}=1.31-1.317)/b,$$

in which t_1_ represents the starting point of a rapid decline from a relatively stable and sustained high value, and t_2_ represents the starting point of a gradual decline. These two points divide the logistic model into three stages: a stable period of slow decline, also name as a relatively stable period of high CAP (0−t_1_), period of rapid decline (t_1_−t_2_), and period of gradual decline (t_2_−∞).

### Antioxidant enzyme activities and malondialdehyde contents of flag and penultimate leaves

For each treatment, 30 flag and penultimate leaves were collected randomly from 5 to 25 DPA, immediately placed into liquid N, and stored at −80°C for future determination of antioxidant enzyme [superoxide dismutase (SOD), peroxidase (POD), catalase (CAT)] activities and malondialdehyde (MDA) content.

Fresh flag leaves or penultimate leaves (0.5 g) were weighed and placed into a mortar, and 5 mL of phosphate buffer (pH = 7.8) was added. After grinding in an ice bath, the homogenate was poured into a centrifuge tube and then centrifugated at 12,000 × g for 20 min at 4°C. The supernatant (crude enzyme extract) was poured into a tube and stored at 0–4°C until being used for testing the activities of SOD (Giannopolitis and Ries, 1977), POD (Gonçalves et al., 1998), and CAT (Kato and Shimizu, 1987), and the content of MDA (Hodges et al., 1999; Murshed et al., 2008).

### Statistical analysis

Analysis of variance (ANOVA) was carried out with DPS software (version 7.05; Zhejiang University, Hangzhou, China). Means were compared between treatments using the post-hoc least significant difference (LSD) test, *P <* 0.05 was considered significant. Tables were created in Microsoft Excel 2013 (Microsoft Corp., Redmond, WA, United States), and figures were generated in SigmaPlot (version 12.5; Systat Software Inc., San Jose, CA, United States).

## Results

### Grain yield and its components

**FIGURE 3:**
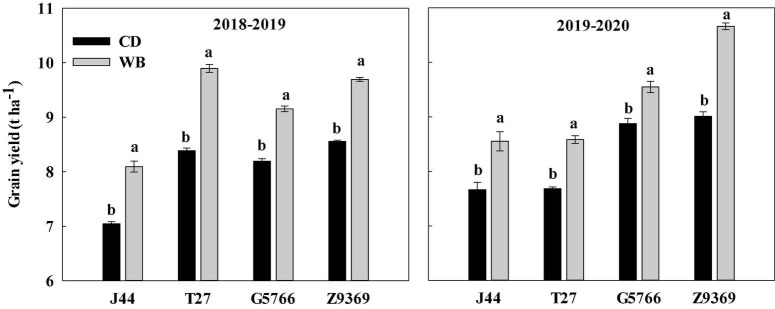
Effects of sowing pattern on the grain yield of bread wheat. Values are means of three replicates per treatment. Vertical bars indicate standard error. Different letters in the same season and cultivar indicate a significant difference at *P* < 0.05, as determined by the LSD test. CD and WB denote the conventional drilling and wide belt sowing pattern, respectively.

The ANOVA showed that the cultivar, sowing pattern, and the cultivar × sowing pattern interaction significantly influenced grain yield (Supplementary Table 2 and Figure 3). On average, the grain yield in the 2019–2020 growing season was only 2.20% higher than in the 2018–2019 growing season. Among the four bread wheat cultivars, Z9369 had the highest grain yield (9.48 t ha^–1^), followed by G5766 (8.94 t ha^–1^), T27 (8.64 t ha^–1^), and J44 (7.84 t ha^–1^), across the two growing seasons and sowing patterns.

When the sowing pattern was changed from CD to WB, the grain yield of each bread wheat cultivars increased by varying amounts. Compared with CD, the grain yields in WB of J44, T27, G5766, and Z9369 were higher, by 14.89, 17.98, 11.72, and 13.29%, in the 2018–2019 growing season, and by 11.61, 11.72, 7.57, and 18.31% in the 2019–2020 growing season, respectively (Figure 3).

The spike number of the J44, T27, G5766, and Z9369 bread wheat cultivars increased from CD to WB by 11.25, 9.86, 8.80, and 12.14%, respectively. The grain per spike trends of the four cultivars differed between the two sowing patterns in the two growing seasons. However, the grains per unit area were remarkably higher in WB than CD, by 12.25, 14.36, 9.99, and 15.55% for J44, T27, G5766, and Z9369, respectively. The grain weight of each bread wheat cultivar remained unchanged between sowing patterns (Table 2). These indicated that WB increased grain yield mainly due to the increase in the grains per unit area and the maintenance of grain weight.

**TABLE 2:** Effects of sowing pattern on grain yield and components of bread wheat.

Growing season	Cultivar	Sowing pattern	Spike number	Grains per spike	Grains per unit area	Grain weight
			(10^4^ ha^–1^)		(10^4^ m^–2^)	(mg)
2018–2019	J44	CD	522.00b	35.17a	1.84b	44.13a
		WB	607.11a	34.47a	2.09a	44.47a
	T27	CD	571.78b	42.30b	2.42b	39.87a
		WB	651.55a	43.58a	2.84a	40.06a
	G5766	CD	600.67b	41.75a	2.51b	37.57a
		WB	680.89a	41.35a	2.82a	37.38a
	Z9369	CD	490.89b	53.07a	2.61b	37.77a
		WB	552.00a	53.65a	2.96a	37.63a
2019–2020	J44	CD	733.14b	31.19b	2.29b	38.53a
		WB	778.63a	32.62a	2.54a	38.73a
	T27	CD	608.82b	44.78b	2.73b	32.40a
		WB	643.89a	47.29a	3.04a	32.42a
	G5766	CD	812.75b	32.23b	2.62b	38.96a
		WB	847.25a	33.24a	2.82a	38.97a
	Z9369	CD	769.22b	33.84b	2.60b	39.79a
		WB	860.20a	35.62a	3.06a	39.99a

Values followed by different letters within a column with the same season and cultivar indicate a significant difference at P < 0.05, as determined by the LSD test. CD and WB denote the conventional drilling and wide belt sowing pattern, respectively.

### Pre- and post-anthesis dry matter remobilization and production

The ANOVA showed that the cultivar, sowing pattern, and cultivar × sowing pattern interaction significantly influenced DM at anthesis and maturity, while only the cultivar had a significant influence on the harvest index (HI) (Supplementary Table 2). From CD to WB, the DM at anthesis and maturity of each bread wheat cultivar increased by varying amounts, but the HI was unchanged (Supplementary Figures 1, 2). The sowing pattern also had a significant influence on DMRA, DMRE, DMRCR, DMPA, and DMACR (Table 3). On average, DMRA, DMRE, and DMRCR were higher in the 2018–2019 growing season than 2019–2020 growing season. The opposite was true for DMPA and DMACR, which were higher in the 2019–2020 growing season than in the 2018–2019 growing season. In a comparison of the four bread wheat cultivars, the DMRA, DMRE, DMRCR, DMPA, and DMACR values of J44, T27, G5766, and Z9369 were completely different across the two growing seasons and sowing patterns.

**TABLE 3:** Effects of sowing pattern on the dry matter remobilization, production, and contribution rate to grain dry matter of bread wheat.

Growing season	Cultivar	Sowing pattern	DMRA	DMRE	DMRCR	DMPA	DMACR
			
			(t ha^–1^)	(%)	(%)	(t ha^–1^)	(%)
2018–2019	J44	CD	2.84a	27.48a	35.07a	5.26b	64.93b
		WB	2.84a	24.24b	30.47b	6.47a	69.53a
	T27	CD	2.14a	19.59a	22.17a	7.50b	77.83b
		WB	1.81b	14.24b	15.94b	9.56a	84.06a
	G5766	CD	2.63a	22.71a	27.91a	6.79b	72.09b
		WB	2.75a	21.58b	26.12b	7.77a	73.88a
	Z9369	CD	2.36a	20.12a	24.02a	7.47b	75.98b
		WB	2.54a	19.32b	22.79b	8.60a	77.21a
2019–2020	J44	CD	1.93a	13.51a	21.90a	6.88b	78.10b
		WB	1.31b	8.65b	13.34b	8.52a	86.66a
	T27	CD	2.06a	15.03a	23.27a	6.78b	76.73b
		WB	1.75b	11.76b	17.77b	8.11a	82.23a
	G5766	CD	2.05a	14.61a	20.08a	8.16b	79.92b
		WB	1.83b	12.61b	16.68b	9.15a	83.32a
	Z9369	CD	2.57a	18.21a	24.73a	7.79b	75.27b
		WB	2.47a	16.04b	20.19b	9.78a	79.81a
Growing season (S)			104.17***	487.98***	605.32***	94.01***	142.01***
Cultivar (C)			22.29***	22.33***	87.99***	89.17***	20.64***
Sowing pattern (P)			10.99**	73.35***	359.33***	365.01***	84.30***
S × C			39.03***	70.53***	256.19***	74.78***	60.10***
S × P			9.99**	0.45	18.57***	0.92	4.36*
C × P			3.59*	5.32**	18.53***	4.31*	4.35*
S × C × P			1.85	1.65	4.90**	5.22**	1.15

Values followed by the same letter within a column in the same year and wheat cultivar are not significantly different at P < 0.05, as determined by the least significant difference test. Values are means of three replicates per treatment. *, **, and ***Indicate significance at the 0.05, 0.01, and 0.001 probability levels, respectively. CD and WB denote the conventional drilling and wide belt sowing pattern, respectively.

DMRA, dry matter remobilization amount in vegetative organs; DMRE, dry matter remobilization efficiency in vegetative organs; DMRCR, total contribution rate of pre-anthesis dry matter remobilization to yield; DMPA, post-anthesis dry matter production amount; DMACR, total contribution ratio of post-anthesis dry matter accumulation to grain.

From CD to WB, the DMRA of each bread wheat cultivar decreased slightly or remained unchanged, while DMRE and DMRCR both decreased. The DMRE values of J44, T27, G5766, and Z9369 decreased by 23.87, 24.52, 9.32, and 7.95%, respectively, and the DMRCR values of J44, T27, G5766, and Z9369 decreased by 26.09, 25.88, 11.69, and 11.74% across the two growing seasons. DMPA and DMACR were increased significantly in WB in comparison to CD. For J44, T27, G5766, and Z9369, the DMPA increased by 23.44, 23.58, 13.33, and 20.33%, and the DMACR by 9.02, 7.59, 3.37, and 3.82%, respectively, across the two growing seasons. Compared with CD, the WB improved the grain DM of bread wheat mainly by increasing DMPA.

### Canopy apparent photosynthesis

#### Dynamic changes in canopy apparent photosynthesis and logistic regression model

**FIGURE 4:**
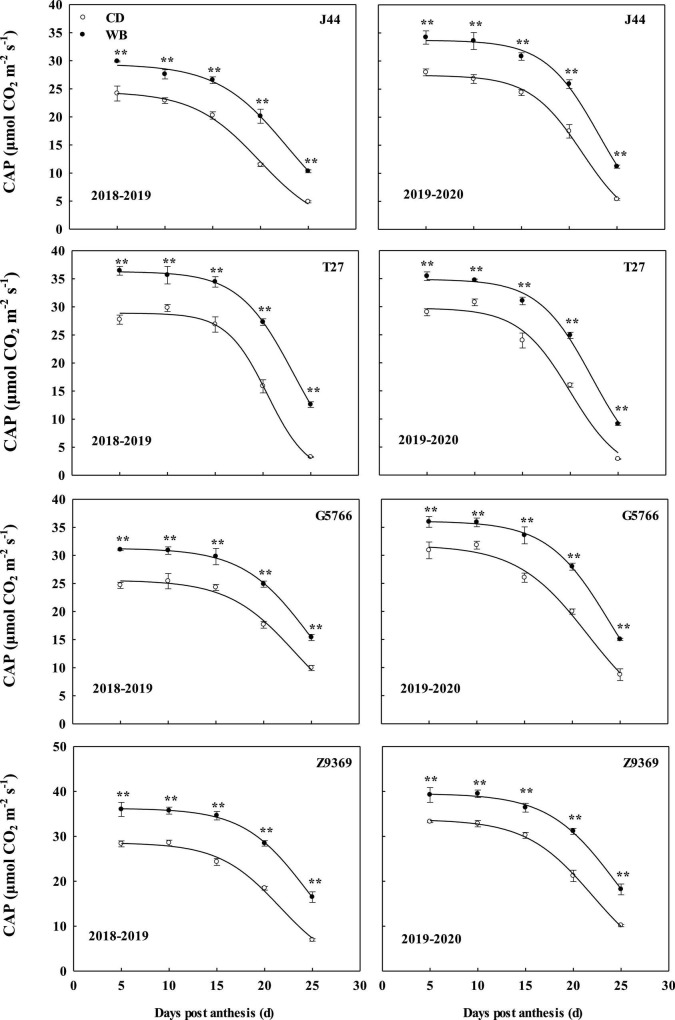
Effects of sowing pattern on the canopy apparent photosynthesis (CAP) of bread wheat. Values are means of three replicates per treatment. Vertical bars indicate standard error. **Indicates significant differences at *P* < 0.01 between the two sowing patterns at each measurement time. CD and WB denote the conventional drilling and wide belt sowing pattern, respectively.

The dynamic changes in CAP and model parameters of the four bread wheat cultivars are shown in Figure 4 and Table 4. The sowing pattern significantly affected the CAP of the bread wheat cultivars (Figure 4), which was higher in WB than CD from 5 to 25 DPA. On average, the CAP values of J44, T27, G5766, and Z9369 were higher by 22.83–109.84, 16.20–253.00, 17.16–63.41, and 22.51–108.48%, respectively.

**TABLE 4:** Effect of sowing pattern on the fitting of canopy apparent photosynthesis (CAP) model parameters of bread wheat post-anthesis.

Growing season	Cultivar	Sowing pattern	Duration of relatively stable CAP post-anthesis (T)	Highest theoretical CAP value (*K*)	*P*
2018–2019	J44	CD	15.34	24.48	<0.01
		WB	17.98	29.41	<0.01
	T27	CD	17.64	28.88	<0.01
		WB	19.41	36.29	<0.001
	G5766	CD	18.66	25.59	<0.05
		WB	20.26	31.28	<0.001
	Z9369	CD	17.39	28.55	<0.001
		WB	20.02	36.22	<0.001
2019–2020	J44	CD	17.74	27.46	<0.01
		WB	19.40	33.71	<0.01
	T27	CD	16.44	29.75	<0.05
		WB	18.56	34.88	<0.01
	G5766	CD	16.73	31.80	<0.05
		WB	19.64	36.11	<0.001
	Z9369	CD	17.42	33.75	<0.01
		WB	19.81	39.53	<0.01

The p-value reflects the goodness of fit of the three-parameter logistic regression.

CD and WB denote the conventional drilling and wide belt sowing pattern, respectively.

Post-anthesis CAP first showed a relatively stable period of high CAP (from anthesis to t_1_), followed by a rapid fall (t_1_ to t_2_), and finally a slow decline (Figure 4). Based on the simulated model of CAP, Z9369 had the highest theoretical maximum (K, the theoretically highest value of CAP), followed by T27, G5766, and finally J44, across the two growing seasons and sowing patterns.

As shown in Table 4, compared with CD, the highest theoretical CAP values of J44, T27, G5766, and Z9369 in WB were increased by 21.45, 21.45, 17.89, and 22.00%, across the two growing seasons. And the time at which the CAP declined rapidly was delayed by 2.15, 1.95, 2.26, and 2.51 days for J44, T27, G5766, and Z9369, respectively, across the two growing seasons, indicating that WB prolonged the duration of relatively stable CAP.

#### Correlation analysis

**FIGURE 5:**
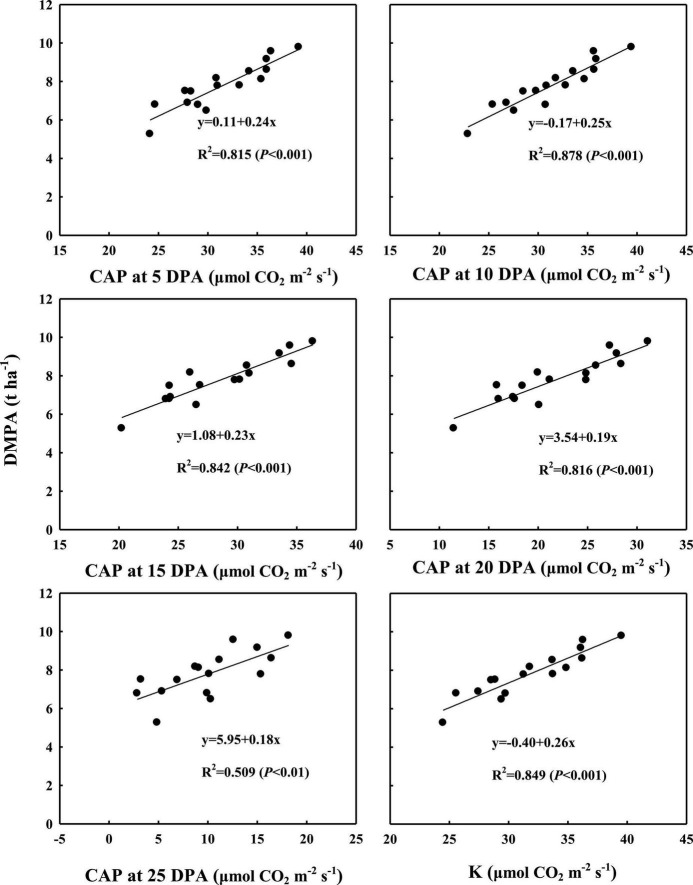
Correlation analyses between dynamic changes in canopy apparent photosynthesis (CAP) from 5 days post-anthesis (DPA) to 25 DPA and the highest theoretical CAP value (K) with dry matter amount post-anthesis (DMPA). Values are means of three replicates per treatment.

**FIGURE 6:**
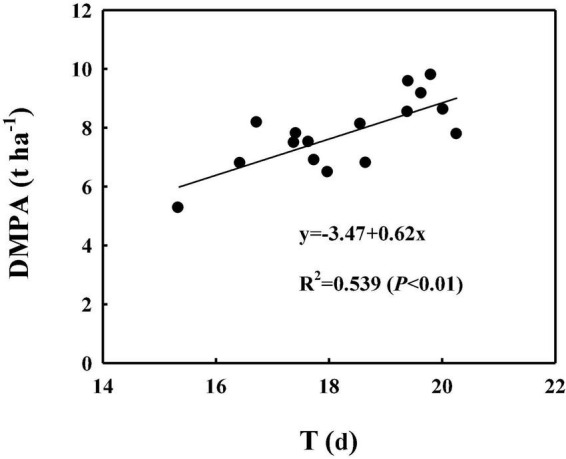
Correlation analyses between the theoretical duration of the period of relatively stable CAP post-anthesis (from anthesis to the time at which the CAP declined rapidly, T) with the dry matter production amount post-anthesis (DMPA). Values are means of three replicates per treatment.

As shown in Figures 5, 6, the correlation analysis showed that the DMPA was linearly and positively related to dynamic changes in CAP from 5 to 25 DPA, the highest CAP value (K) and duration of the relatively stable CAP post-anthesis (from anthesis to the time at which the CAP became fast falling, T). These demonstrated that WB improved DMPA compared with CD mainly through increasing the rate of CAP, delaying the time at which the CAP declined rapidly, and increasing the duration of the period of relatively stable CAP post-anthesis.

### Antioxidant enzyme activities and malondialdehyde content of flag leaf and penultimate leaf

#### Superoxide dismutase enzyme activities of flag leaf and penultimate leaf

**FIGURE 7:**
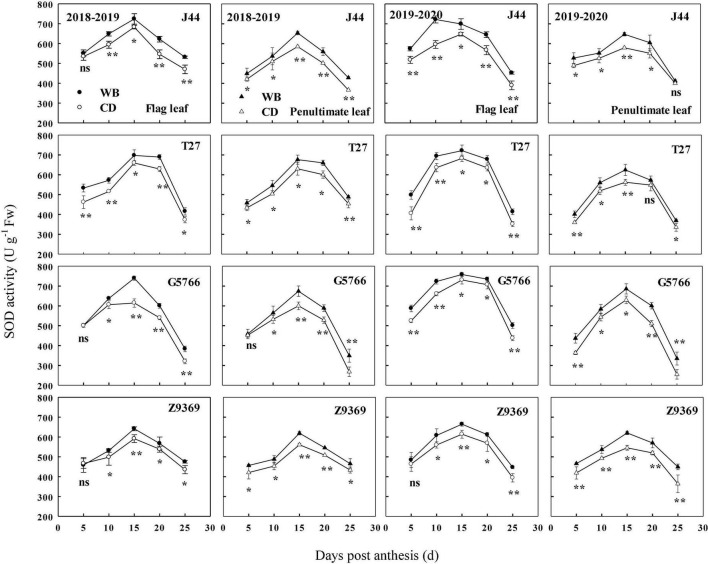
Effects of sowing pattern on superoxide dismutase (SOD) activities of the flag leaf and penultimate leaf of bread wheat. Values are means of three replicates per treatment. Vertical bars indicate standard error. ns, *, and **Indicate significant differences at *P* ≥ 0.05, *P* < 0.05, and *P* < 0.01 between the two sowing patterns at each measurement time. CD and WB denote the conventional drilling and wide belt sowing pattern, respectively.

The sowing pattern significantly affected the SOD enzyme activities of the flag leaf and penultimate leaf of bread wheat (Figure 7). The SOD enzyme activities first increased and then decreased from anthesis to maturity. From 0 to 15 DPA, the SOD enzyme activities of the bread wheat cultivars increased and reached their highest value. At 15 DPA, J44 had the highest SOD activity for flag leaf, followed by T27, G5766, and Z9369, across two growing seasons. For the penultimate leaf, the ranking, in descending order, was G5766, J44, T27, and Z9369, respectively. The values all decreased after 15 DPA. During the whole grain filling period, the SOD enzyme activities of the flag leaf and penultimate leaf of the bread wheat cultivars sown with WB were higher than or equal to those of CD. On average, the SOD enzyme activities of the flag leaf of J44, T27, G5766, and Z9369 were higher by 6.87–15.19, 5.74–19.10, 7.47–19.29, and 1.40–11.24%, while those of the penultimate leaf were higher by 5.15–11.74, 7.16–9.18, 6.72–30.72, and 8.22–15.40%, respectively, across the two growing seasons.

#### Peroxidase enzyme activities of flag leaf and penultimate leaf

**FIGURE 8:**
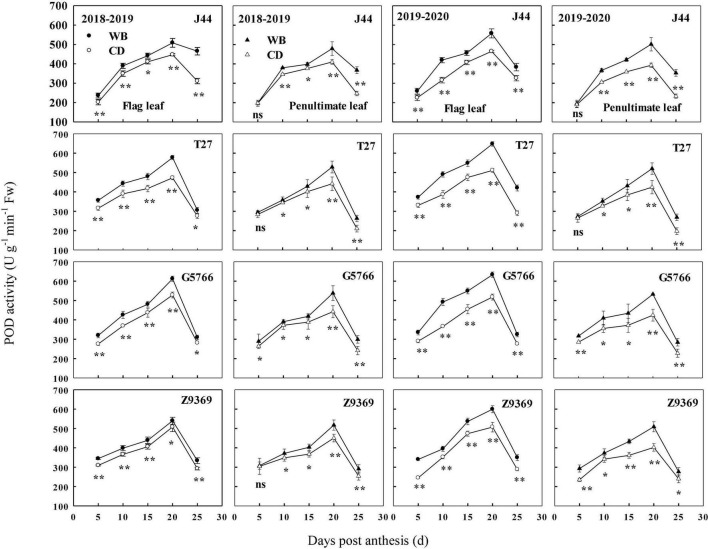
Effects of sowing pattern on peroxidase (POD) activities of the flag leaf and penultimate leaf of bread wheat. Values are means of three replicates per treatment. Vertical bars indicate standard error. ns, *, and **Indicate significant differences at *P* ≥ 0.05, *P* < 0.05, and *P* < 0.01 between the two sowing patterns at each measurement time. CD and WB denote the conventional drilling and wide belt sowing pattern, respectively.

The sowing patterns significantly affected the POD enzyme activities of the flag leaf and penultimate leaf of bread wheat (Figure 8). POD enzyme activities first increased and then decreased from anthesis to maturity, reaching their highest values at about 20 DPA. G5766 had the highest POD activity of flag leaf at 20 DPA, followed by T27, Z9369, and J44, across two growing seasons. For the penultimate leaf, the ranking, in descending order, was G5766, T27, Z9369, and J44, respectively. All values decreased after 20 DPA. During the whole grain filling period, the POD enzyme activities of the flag leaf and penultimate leaf of the bread wheat cultivars sown in WB were higher than or equal to those of CD. On average, the POD enzyme activities of the flag leaf of J44, T27, G5766, and Z9369 were higher by 9.73–33.55, 12.76–27.89, 13.77–24.86, and 10.50–25.08%, and those of the penultimate leaf were increased by 1.39–50.24, 3.34–30.59, 10.00–24.35, and 7.92–20.62%, respectively, across the two growing seasons.

#### Catalase enzyme activities of flag leaf and penultimate leaf

**FIGURE 9:**
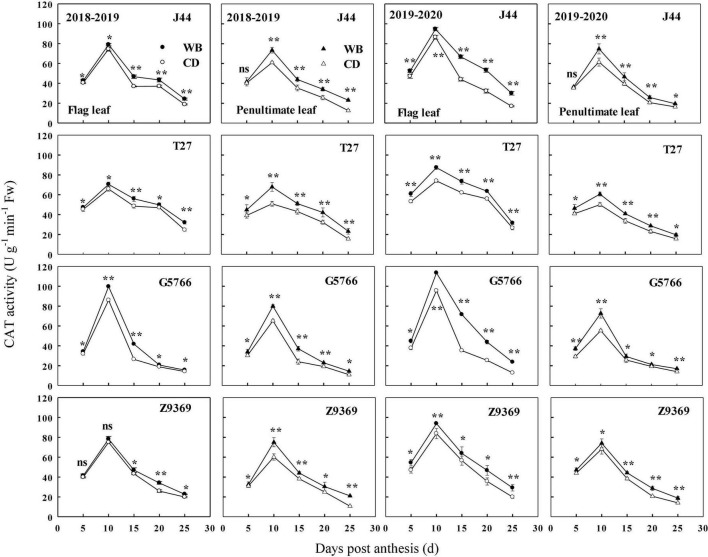
Effects of sowing pattern on catalase (CAT) activities of the flag leaf and penultimate leaf of bread wheat. Values are means of three replicates per treatment. Vertical bars indicate standard error. ns, *, and **Indicate significant differences at *P* ≥ 0.05, *P* < 0.05, and *P* < 0.01 between the two sowing patterns at each measurement time. CD and WB denote the conventional drilling and wide belt sowing pattern, respectively.

The sowing patterns significantly affected CAT enzyme activities of the flag leaf and penultimate leaf of the bread wheat (Figure 9). The CAT enzyme activities first increased and then decreased from anthesis to maturity. From 0 to 10 DPA, the CAT enzyme activities of the bread wheat cultivars increased to their highest values. At 10 DPA, G5766 had the highest CAT activity for flag leaf, followed by J44, Z9369, and T27, across two growing seasons. For the penultimate leaf, the ranking, in descending order, was G5766, Z9369, J44, and T27, respectively. All values decreased after 10 DPA. During the whole grain filling period, the CAT enzyme activities of the flag leaf and penultimate leaf of the bread wheat cultivars sown in WB were higher than or equal to those sown in CD. On average, the CAT enzyme activities of the flag leaf of J44, T27, G5766, and Z9369 were higher by 7.78–53.00, 9.33–24.56, 12.90–80.81, and 8.23–31.42%, and those of the penultimate leaf were increased by 2.91–51.24, 13.78–37.10, 15.03–34.04, and 6.11–66.78%, respectively, across the two growing seasons.

#### Malondialdehyde contents of flag leaf and penultimate leaf

**FIGURE 10:**
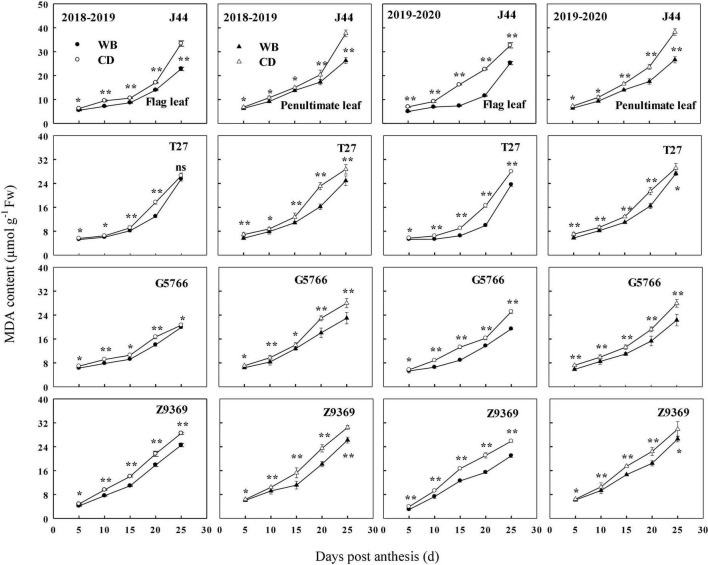
Effects of sowing pattern on malondialdehyde (MDA) content of flag leaf and penultimate leaf of bread wheat. Values are means of three replicates per treatment. Vertical bars indicate standard error. * and **Indicate significant differences at *P* < 0.05 and *P* < 0.01 between the two sowing patterns at each measurement time, respectively. CD and WB denote the conventional drilling and wide belt sowing pattern, respectively.

The sowing pattern significantly affected the MDA contents of the flag leaf and penultimate leaf of the bread wheat (Figure 10). The MDA content increased from anthesis to maturity. However, the MDA contents of the flag leaf and penultimate leaf of the bread wheat cultivars sown in WB were lower than those sown in CD. On average, the MDA contents of the flag leaf of J44, T27, G5766, and Z9369 were diminished by 19.87–36.28, 6.99–33.34, 7.72–22.88, and 16.37–23.34%, and those of the penultimate leaf were decreased by 9.76–30.24, 9.88–26.64, 13.03–20.77, and 5.21–21.58%, respectively, across the two growing seasons.

In brief, WB can increase antioxidant capacity by increasing antioxidant enzyme activities and decreasing the MDA contents of the flag leaf and penultimate leaf of the bread wheat cultivars during the whole grain filling period. All of these were beneficial to the extension of the duration of stable CAP.

## Discussion

Similar to the results of previous studies on common wheat (Li et al., 2015; Fan et al., 2019), improvements in grain yield of four bread wheat cultivars in comparison to CD were observed in WB. This constitutes additional evidence that optimizing seedling belt width can improve the grain yield of bread wheat, and that WB can be applied widely in wheat production.

Wheat yield is frequently analyzed in terms of yield components, i.e., spike number, grains per spike, and grain weight (Duggan et al., 2000; Sadras and Slafer, 2012; Li et al., 2019). The spike number and grains per spike determine the grains per unit area. Therefore, the yield could be considered a consequence of the grains per unit area produced by the crop and average weight of these grains. Naturally, increases in either of these two components would increase yield (Slafer et al., 2022). Consistent with the results of most previous reports (Slafer et al., 2014; Fan et al., 2019; Jiao et al., 2022), in the present study WB improved the grain yield of the four bread wheat cultivars in comparison with CD, mainly through increasing spike number and in turn grains per unit area. Nevertheless, we did not find negative trade-offs between grains per unit area and grain size, and the grain weight was maintained in WB. These results demonstrated that WB distributed large amounts of DM to the grain, ensuring large grain size under the condition of increased grains per unit area, consequently improving grain yield.

The DM allocated to grain was affected by the DMRA and DMPA (Plaut et al., 2004). DMRA can play a buffering role and decrease negative environmental effects during grain filling on final grain yield (Ma et al., 2015; Liu Y. et al., 2020). DMPA is the key factor determining the yield of wheat grains (Arduini et al., 2006; Ercoli et al., 2008; Zhang et al., 2016) because almost all of it is transported to the grain (Austin et al., 1980). Within a certain range, there is an obvious positive correlation between wheat yield and photosynthate production post-anthesis (Zhang et al., 2012). In the present study, despite the equal or slightly lower DMRA, the remarkable increment in DMPA in WB ensured DM distribution to the grain of each bread wheat cultivar, and consequently maintained grain weight.

The DM production of field crops depends on the rate and duration of CAP (Lawson et al., 2012; Carmo-Silva et al., 2017; Chen et al., 2017; Yang et al., 2022). In the case of wheat, a close relationship between DM accumulation and the rate and duration of CAP has been reported (Kruger and Volin, 2006; Parry et al., 2010; Xie et al., 2016). Previous studies showed that WB significantly increased tiller and spike numbers (Li et al., 2015; Fan et al., 2019), leaf area index (Liu et al., 2017), and photosynthetically active radiation capture (Zhao et al., 2013) in comparison with CD. All of these changes enhance the CAP of winter wheat. In our study, the positive linear relationship of DMPA with CAP from 5 to 25 DPA, and with the highest theoretical CAP value (K), confirmed this conclusion, demonstrating that WB leads to remarkable DMPA improvement through an increase in the canopy photosynthetic rate. DMPA was also linearly and positively related to the duration of the period of relatively stable CAP post-anthesis (from anthesis to the time at which the CAP declined rapidly, T), indicating that, in comparison with CD, the longer duration of the period of relatively stable CAP also accounted for DMPA improvement in WB. This was inconsistent with previous studies of wheat (Christopher et al., 2008; Bogard et al., 2011; Derkx et al., 2012) and other crops (Gregersen et al., 2013). However, the mechanism through which WB extended the duration of high canopy photosynthesis remains to be elucidated.

The leaves are the main plant part through which wheat receives light energy and performs photosynthesis (Man et al., 2017). Photosynthesis can vary with leaf age, position, and surface, and among general plant and development stages (Choudhury, 2000). Nevertheless, during grain filling, leaf photosynthesis gradually decreases as the leaves begin senescence (Man et al., 2017). Longer functional photosynthesis with postponed leaf senescence could result in greater accumulation of photosynthates, thereby enhancing crop productivity and ultimately increasing wheat yields (Kim et al., 2011; Xie et al., 2016). However, levels of free radicals and concentrations of reduced oxygen species, such as hydrogen peroxide, increase during senescence (Merzlyak and Hendry, 1994). Wheat deploys antioxidases (such as SOD, POD, and CAT) and osmotic-adjustment substances to scavenge reactive oxygen species (ROS) and free radicals, thereby regulating oxidation processes, protecting the cell from oxidative damage and delaying leaf senescence (Lv et al., 2017). A deficiency in N uptake post-anthesis always increases the production of ROS in plants, which results in lipid peroxidation of cell membranes (Stoparić and Maksimovic, 2008), accelerates leaf senescence, and reduces post-anthesis DM assimilation (Wu et al., 2018; Nehe et al., 2020).

Winter wheat leaves, especially those in the upper part of the canopy, are the sites of most of the photosynthesis that occurs in plants (Zhan et al., 2015). The flag leaf and penultimate leaf, as the topmost two leaves, are the most important leaves for wheat plant photosynthesis (Townsend et al., 2017). In our study, the relatively higher activities of SOD, POD, and CAT, and lower MDA content, of flag leaf and penultimate leaf in WB improved the leaves’ ability to scavenge ROS and free radicals, delayed their senescence, and maintained their function compared to CD. The improved anti-senescence ability in WB may be related to the significantly increased N absorption post-anthesis (Liu et al., 2022).

**FIGURE 11:**
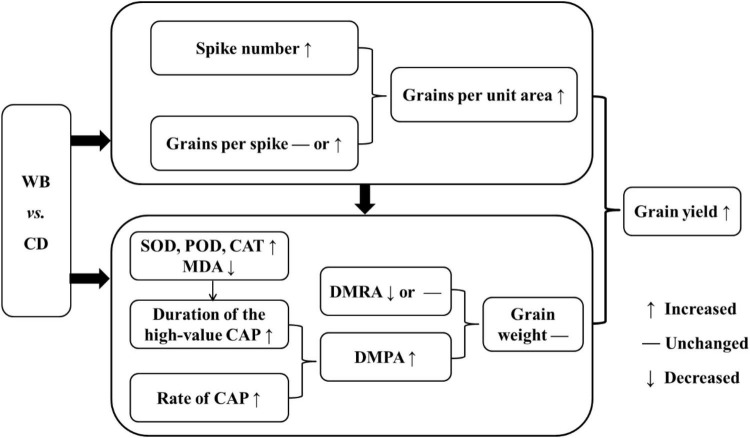
The model of increased grain yield in wide belt sowing (WB) when compared with conventional drilling sowing (CD). SOD, superoxide dismutase; POD, peroxidase; CAT, catalase; MDA, malondialdehyde; CAP, canopy apparent photosynthesis; DMRA, dry matter remobilization amount in vegetative organs; DMPA, post-anthesis dry matter production amount.

Delayed leaf senescence can result in a longer photosynthesis period (Benbella and Paulsen, 1998), greater accumulation of photosynthates for grain filling, and higher wheat yields (Spano et al., 2003; Kim et al., 2011; Evans, 2013; Kromdijk et al., 2016; Xie et al., 2016). In our study, in comparison with CD, WB increased antioxidant enzyme activities, inhibited leaf senescence, prolonged the green leaf period, improved CAP, increased DMPA, and maintained grain weight against a backdrop of increased grains per unit area, thereby ultimately increasing the grain yield of bread wheat (Figure 11).

## Conclusion

Increasing the seedling belt width from 2–3 cm (CD) to 8–10 cm (WB) significantly increased the activities of SOD, POD, and CAT, and decreased the MDA content of the flag and penultimate leaves of bread wheat. In turn, this inhibited leaf senescence and extended the duration of the high-value CAP period. In combination with the increased rate of CAP, a remarkable increment in DMPA was observed in WB, ensuring that the DM was distributed to grains of each bread wheat cultivar and hence maintaining grain weight against a backdrop of increase in spike number and in turn grains per unit area. Consequently, grain yield was significantly improved in WB. In summary, WB can be applied widely to increase the yield in bread wheat production.

## Data availability statement

The original contributions presented in this study are included in the article/Supplementary material, further inquiries can be directed to the corresponding author/s.

## Author contributions

ZJ and XD designed the experiments, managed the projects, and guided the writing of the article. XZ, YH, and YL performed the experiments. XZ performed the data analysis and wrote the manuscript. MH gave useful suggestions during the process of this article. All authors contributed to the article and approved the submitted version.
