# Supplementary material for: Wide belt sowing improves the grain yield of bread wheat by maintaining grain weight at the backdrop of increases in spike number
Source: Front Plant Sci. 2022 Aug 18;13:992772. doi: 10.3389/fpls.2022.992772 (PMC9433909; doi:10.3389/fpls.2022.992772)
Supplement: Supplementary file 1 [file Data_Sheet_1.docx]

**Supplementary information**

Table S1. Nutrient status of top 0-20 cm soil before seeding in 2018-2019 and 2019-2020 growing seasons.

| Growing season | Bulk density  (g cm^–3^) | Organic matter  (g kg^–1^) | Total nitrogen (g kg^–1^) | Alkali-hydrolysable nitrogen  (mg kg^–1^) | Available phosphorus  (mg kg^–1^) | Available potassium  (mg kg^–1^) |
| --- | --- | --- | --- | --- | --- | --- |
| 2018-2019 | 1.31 | 17.80 | 1.12 | 84.71 | 27.84 | 93.72 |
| 2019-2020 | 1.31 | 16.41 | 1.14 | 74.60 | 30.86 | 81.61 |

Table S2. Analysis of variance of grain yield, dry matter (DM) at anthesis, DM at maturity, and harvest index (HI) according to growing season, cultivar, sowing pattern, and their interactions.

| Factor | Grain yield | DM at anthesis | DM at maturity | HI |
| --- | --- | --- | --- | --- |
| Growing season (S) | 16.32*** | 902.80*** | 545.28*** | 501.29*** |
| Cultivar (C) | 201.58*** | 12.12*** | 46.76*** | 20.46*** |
| Sowing pattern (P) | 514.50*** | 189.12*** | 307.22*** | 0.10 |
| S × C | 69.52*** | 16.35*** | 34.62*** | 26.77*** |
| S × P | 1.96 | 6.31* | 2.74 | 1.67 |
| C × P | 6.95** | 3.03* | 3.96* | 1.21 |
| S × C × P | 5.95** | 0.69 | 1.26 | 0.08 |

*, **, and *** indicate significance at the 0.05, 0.01, and 0.001 probability levels, respectively.

Fig. S1 Effects of sowing pattern on dry matter accumulation at anthesis and maturity of bread wheat. Values are means of three replicates per treatment. Vertical bars indicate standard error. Different letters with the same season and cultivar indicate significant differences at *P* < 0.05, as determined by the LSD test. CD and WB denote the conventional drilling and wide belt sowing pattern, respectively.

Fig. S2 Effects of sowing pattern on the harvest index of bread wheat. Values are means of three replicates per treatment. Vertical bars indicate standard error. Different letters with the same season and cultivar indicate significant differences at *P* < 0.05, as determined by the LSD test. CD and WB denote the conventional drilling and wide belt sowing pattern, respectively.
